# Tardy posterior interosseous nerve palsy associated with radial head fracture: a case report

**DOI:** 10.1186/1757-1626-2-22

**Published:** 2009-01-07

**Authors:** Jas Daurka, Alvin Chen, Kashif Akhtar, Srinath Kamineni

**Affiliations:** 1 Department of Orthopaedic Surgery, 7 East, Charing Cross Hospital, Fulham Palace Road, Hammersmith, London, UK

## Abstract

**Background:**

A 55 year old gentleman presented with tardy posterior interosseous nerve palsy associated with radial head fracture.

**Case presentation:**

The patient developed symptoms of wrist drop 24 hours after the injury whilst awaiting surgery for his Mason III radial head fracture. EMG studies confirmed the presence of a posterior interosseous nerve lesion. Open exploration revealed oedematous soft tissues surrounding the nerve. The proximity of the nerve to the fracture and its course through the arcade of Frohse make it susceptible to injury, from the initial traumatic event and the following oedema.

**Conclusion:**

A full recovery occurred after exploration and decompression of the nerve.

## Case presentation

A 55 year old Caucasian manual worker presented to the emergency department after a fall on his outstretched right hand. He was of average build and denied a smoking history. He denied a significant medical or family history, and was not on any medication.

A Mason III radial head fracture was diagnosed and the patient was initially managed with a broad arm sling, with an appointment to return for orthopaedic review the following day. His initial neurological examination was entirely normal. He was subsequently seen and assessed in the orthopaedic out-patient fracture clinic 24 hours after his initial injury. Preliminary examination findings revealed a loss of extension and supination at the elbow joint of 20° and 10° respectively. There was no evidence of a neurological deficit at this stage, with normal wrist and finger extension and first dorsal web-space sensation. In an attempt to improve functional range of movement, an arthroscopic exploration and possible reduction and fixation of the fracture fragments was recommended.

Whilst awaiting surgery, 36 hours following the initial injury he developed wrist finger and thumb drop with no associated sensory impairment. Electromyographic (EMG) studies at this stage confirmed the presence of a partial posterior interosseous nerve (PIN) palsy. An MRI scan revealed signal changes in keeping with oedema surrounding the superior radio ulnar joint with no evidence of encroachment by fracture haematoma. The interosseous membrane was visualized and noted to show no evidence of acute injury.

**Figure 1:**
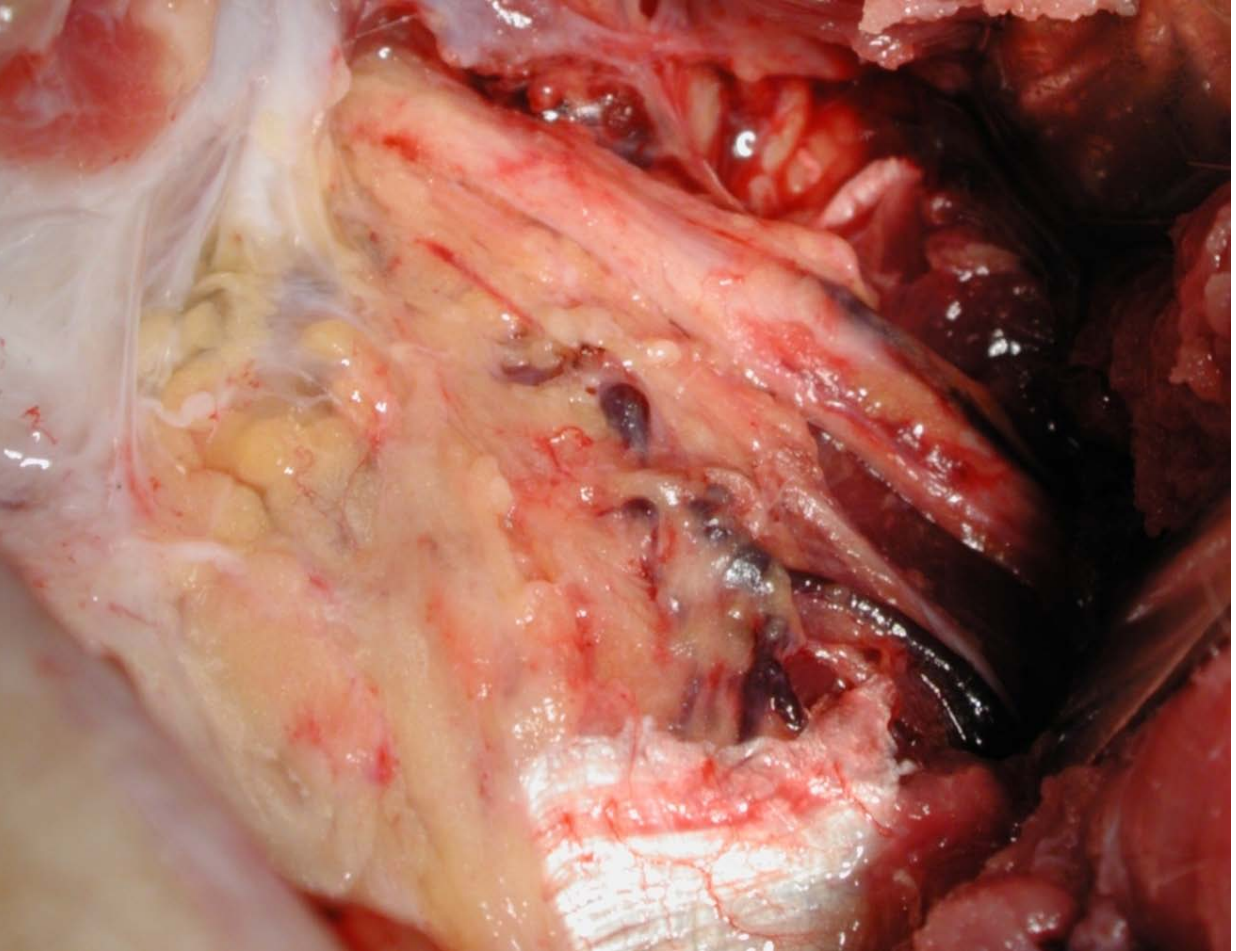
**A completely decompressed Posterior Interosseous Nerve with resection of the oedamatous soft-tissues**. Peri-neural blood vessels are seen to have re-appeared.

Arthroscopic reduction could not be performed due to extensive fracture comminution and hence a partial excision of the radial head fragments causing a block to the patients range of motion was performed, leaving 60% of the articular surface of the radial head in stable continuity with the radial neck. Intra-operative stability examination revealed a stable valgus stress test in both full extension and 30 degrees of flexion. Open exploration of the posterior interosseous nerve was performed by a direct anterolateral approach, brachioradialis muscle belly splitting. The interface soft-tissues between the mobile wad musclulature and the supintaor were intact, with mild swelling of the deeper muscle layers. The supinator muscle was also mildly oedematous but structurally intact. The radial nerve was identified proximal to the supinator tunnel and noted to be essentially normal. The supinator, superficial layer, was incised and the PIN was dissected free of the odematous surrounding space – occupying tissues. The nerve, itself, was oedematous but structurally in continuity, with an absence of visible perineural blood vessels. The nerve was completely released and neurolysed throughout the entire extent of the supinator tunnel, following which the peri-neural vessels were seen to re-appear (figure 1).

Following a 6 week period of rehabilitation the PIN function recovered to normal, corroborated by further EMG studies. Elbow range of motion at final follow-up (12 months) was 10 degrees extension deficit, 140 degrees of flexion.

## Discussion

An extensive literature review failed to reveal any previously reported cases of a tardy injury to the posterior interosseous nerve associated with radial head fracture. Traumatic injury of the nerve has been associated with radial head dislocation, radius and ulna diaphyseal fractures, penetrating injuries and contusions[1]. A variety of non traumatic causes including ganglia[2], lipoma[3], rheumatoid synovitis[4] and septic arthritis[5] have also been attributed as a cause of palsy.

The proximity of the PIN to the radiocapitellar joint makes it vulnerable in traumatic injuries around the elbow. A cadaveric study on 50 specimens identified the average distance from the radiocapitellar joint to the origin of the PIN as 1.2 mm +/- 1.9 mm with take off being proximal in 31(62%) and distal in 19(48%). In 49(98%) specimens the PIN remained intramuscular and only in 1(2%) did the nerve come into contact with the radius[6].

Examination of the patient revealed a Type I injury of the PIN in which the function of both descending and recurrent branches was compromised indicating a lesion proximal to its exit from the supinator muscle[7].

This site corresponds to the nerves path through the arcade of Frohse, which is a common site of entrapment neuropathy[7]. EMG studies suggested the nerve lesion was in continuity. It was decided to perform an MRI which revealed no evidence of a lesion causing a mass effect on the nerve. During exposure of the nerve it was noted that the surrounding soft tissues were oedematous and mildly contused, in keeping with the zone of injury from the fracture.

In injuries involving fractures or dislocation of the radius it has been recommended to explore early if the injury is open, and to avoid exploration if it is closed [9]. This conclusion was based on the experience of the authors who followed 17 patients with traumatic PIN palsy.

## Conclusion

This case reveals the previously unreported complication of tardy PIN palsy associated with radial head fracture. The proximity of the nerve to the fracture area and its course through the arcade of Frohse make it susceptible to injury, both from the initial traumatic event and the subsequent surrounding soft-tissue oedema. Both electromyographic and imaging studies helped identify the site of injury and hence aided pre-operative planning. In this case we opted to explore and decompress the nerve at the time of radial head surgery and the outcome was favourable, with full neurological and elbow functional recovery achieved.

## Consent

Written informed consent was obtained from the patient for publication of this case report and accompanying images. A copy of the written consent is available for review by the Editor-in-Chief of this journal.

## Competing interests

The authors declare that they have no competing interests.

## Authors' contributions

JD admitted and commenced initial treatment plans, SK operated on the patient and KA and AC analyzed and interpreted the patient data with regards to the published literature/current understanding of the disease process.
